# Association of a rare variant of the *TNFSF13B* gene with susceptibility to Rheumatoid Arthritis and Systemic Lupus Erythematosus

**DOI:** 10.1038/s41598-018-26573-4

**Published:** 2018-05-29

**Authors:** David González-Serna, Lourdes Ortiz-Fernández, Sofía Vargas, Antonio García, Enrique Raya, Benjamín Fernández-Gutierrez, Francisco Javier López-Longo, Alejandro Balsa, Isidoro González-Álvaro, Javier Narvaez, Carmen Gómez-Vaquero, José Mario Sabio, Rosa García-Portales, María Francisca González-Escribano, Carles Tolosa, Patricia Carreira, Lambertus Kiemeney, Marieke J. H. Coenen, Torsten Witte, Matthias Schneider, Miguel Ángel González-Gay, Javier Martín

**Affiliations:** 1 Institute of Parasitology and Biomedicine López-Neyra, CSIC, Granada, Spain; 2 Rheumatology Department, Hospital Virgen de las Nieves, Granada, Spain; 3 Systemic Autoimmune Diseases Unit, Hospital Campus de la Salud, Granada, Spain; 4 Rheumatology Service, Hospital Clínico San Carlos, IDISSC, Madrid, Spain; 5 Rheumatology Service, Hospital General Universitario Gregorio Marañón, Madrid, Spain; 6 Rheumatology Department, Instituto de Investigación Hospital Universitario La Paz (IDIPAZ), Madrid, Spain; 7 Rheumatology Department, Instituto de Investigación del Hospital de La Princesa (IIS-IP), Madrid, Spain; 8 Rheumatology Service, Hospital Universitario de Bellvitge, Barcelona, Spain; 9 Systemic Autoimmune Diseases Unit, Department of Internal Medicine, Hospital Virgen de las Nieves, Granada, Spain; 10 Rheumatology Department, Hospital Virgen de la Victoria, Málaga, Spain; 11 Department of Immunology, Hospital Universitario Virgen del Rocío, IBIS, Sevilla, Spain; 12 Department of Internal Medicine, Hospital Parc Taulí, Sabadell, Spain; 13 Rheumatology Service, Hospital Universitario 12 de Octubre, Madrid, Spain; 14 Department for Health Evidence, Radboud Institute for Health Sciences, Radboud university medical center, Nijmegen, The Netherlands; 15 Department of Human Genetics, Radboud Institute for Health Sciences, Radboud university medical center, Nijmegen, The Netherlands; 16 Department for Clinical Immunology and Rheumatology, Hannover Medical School, Hannover, Germany; 17 Policlinic and Hiller Research Unit for Rheumatology, UKD, Heinrich-Heine-University Dusseldorf, Dusseldorf, Germany; 18 Epidemiology, Genetics and Atherosclerosis Research Group on Systemic Inflammatory Diseases IDIVAL, Hospital Universitario Marqués de Valdecilla, Santander, Spain

## Abstract

A rare variant (BAFF-var) of the tumor necrosis factor superfamily 13b (*TNFSF13B*) gene has been recently associated with multiple sclerosis (MS) and systemic lupus erythematosus (SLE). The aim of this study was to investigate the association between *TNFSF13B* BAFF-var and susceptibility to rheumatoid arthritis (RA) and replicate that association in SLE. 6,218 RA patients, 2,575 SLE patients and 4,403 healthy controls from three different countries were included in the study. *TNFSF13B* BAFF-var was genotyped using TaqMan allelic discrimination assay. PLINK software was used for statistical analyses. *TNFSF13B* BAFF-var was significantly associated with RA (p = 0.015, OR = 1.21, 95% CI = 1.03–1.41) in the Spanish cohort. A trend of association was observed in the Dutch (p = 0.115) and German (p = 0.228) RA cohorts. A meta-analysis of the three RA cohorts included in this study revealed a statistically significant association (p = 0.002, OR = 1.24, 95% CI = 1.10–1.38). In addition, *TNFSF13B* BAFF-var was significantly associated with SLE in the Spanish (p = 0.001, OR = 1.41, 95% CI = 1.14–1.74) and the German cohorts (p = 0.030, OR = 1.86, 95% CI = 1.05–3.28), with a statistically significant p-value obtained in the meta-analysis (p = 0.0002, OR = 1.46, 95% CI = 1.09–2.32). The results obtained confirm the known association of *TNFSF13B* BAFF-var with SLE and, for the first time, demonstrate that this variant contributes to susceptibility to RA.

## Introduction

Rheumatoid arthritis (RA) and systemic lupus erythematosus (SLE) are complex autoimmune diseases influenced by both genetic and environmental factors^1,2^. Genome-wide association studies (GWAS) have revealed more than 100 independent signals for RA and 50 for SLE associated with these diseases^3,4^. In a recent study Steri *et al*.^5^ identified an association signal in the *TNFSF13B* gene which was associated with multiple sclerosis (MS) and SLE. *TNFSF13B* encodes tumor necrosis factor superfamily member 13b (also known as B-cell activating factor (BAFF)). BAFF is a cytokine that is primarily produced by monocytes and neutrophils and plays a crucial role in B-cell homeostasis and the regulation of B-cell maturation, differentiation and survival^6^. This cytokine plays a particularly important role in the pathogenesis of SLE, to such an extent that the first targeted therapy approved for SLE was Belimumab, a monoclonal antibody targeting human BAFF^7–9^. The causal variant identified by Steri *et al*.^5^ for this association, “BAFF-var”, is an insertion-deletion in which five nucleotides are replaced by one (GCTGT > A), being this A the risk allele. Persons carrying the A allele have an increased risk for the disease. This risk allele results in a shorter transcript that escapes microRNA inhibition thus leading to an increase in the production of soluble BAFF. Besides, it has been observed that *TNFSF13B* BAFF-var is strongly associated with augmented levels of total IgG and IgM and with reduced monocyte counts^5^. Thus, *TNFSF13B* BAFF-var is considered a shared genetic risk variant for autoimmune diseases based on its association with MS and SLE, and it plays an important role in autoimmune processes^5^. Considering that replication of previous results is of vital importance for the correct development of scientific knowledge, we performed a replication study to corroborate the association of *TNFSF13B* BAFF-var with susceptibility to SLE.

Otherwise, BAFF system dysregulation is involved in the pathogenesis of RA^1,10^ and abnormal levels of BAFF have been detected in serum, synovial fluid and saliva from RA patients^11^. It is noteworthy that more than 10 shared risk loci have been identified for SLE and RA^12,13^. Taking into considerations above, we aimed to assess for the first time the potential association of *TNFSF13B* BAFF-var with RA in patients from Spain, the Netherlands and Germany.

## Materials and Methods

### Study subjects

Overall, 6,218 RA patients and 4,403 healthy controls from Spain, Germany and the Netherlands were enrolled in our study. Regarding the Spanish cohort, DNA was obtained from 4,429 RA patients from the Xeral-Calde University Hospital, Lugo; Marqués de Valdecilla University Hospital, Santander; Bellvitge University Hospital, Barcelona; Virgen del Rocío University Hospital, Seville; Campus de la Salud University Hospital, Granada; and Hospital Clínico San Carlos, La Princesa University Hospital, La Paz University Hospital, 12 de Octubre University Hospital and Gregorio Marañon University Hospital, all located in Madrid. DNA from 3,200 healthy controls was obtained from Banco Nacional de ADN. DNA was obtained from 890 RA patients and 733 controls from the Netherlands at the Radboud University Medical Center (Nijmegen) and the Nijmegen Biomedical Study (NBS)^14^ respectively. DNA was obtained from 890 RA patients and 470 controls of German descent at the Hannover Medical School (Hannover) and Düsseldorf University Hospital. All the patients included in this study were of European descent and had been diagnosed with RA according to the 1987 classification criteria of the American College of Rheumatology^15^.

A total of 2,575 SLE patients from Spain and Germany were included in this study. DNA from 1,160 patients from Spain was obtained at Xeral-Calde University Hospital, Lugo; Virgen del Rocío University Hospital, Sevilla; Virgen de las Nieves University Hospital, Granada; Virgen de la Victoria University Hospital, Málaga, and Parc Taulí University Hospital, Sabadell. DNA from 460 German patients was obtained at the Hannover Medical School, Hannover. All SLE patients were of European descent and fulfilled the American College of Rheumatology criteria for the classification of SLE^16^.

The study was approved by the local ethical committees of the different participating centers (Ethics Committee of the Spanish National Research Council, Xeral-Calde University Hospital, Marqués de Valdecilla University Hospital, Bellvitge University Hospital, Virgen del Rocío University Hospital, Campus de la Salud University Hospital, Hospital Clínico San Carlos, La Princesa University Hospital, La Paz University Hospital, 12 de Octubre University Hospital and Gregorio Marañon University Hospital, Virgen de las Nieves University Hospital, Virgen de la Victoria University Hospital, Parc Taulí University Hospital, Radboud University Medical Center, Hannover Medical School and Düsseldorf University Hospital) in accordance with the tenets of the Declaration of Helsinki. Written informed consent was obtained from all participants prior to enrolment in the study.

### Genotyping

Genomic DNA was isolated from peripheral blood samples using standard salting-out techniques. *TNFSF13B* BAFF-var was genotyped using a TaqMan allelic discrimination custom assay (assay ID: AH0JGPG) (Applied Biosystems, Foster City, California, USA) on an ABI 7900HT Fast Real-Time PCR System (Thermo Fisher). Genotyping call rate was >95% in all sample sets.

### Statistical analysis

All the statistical analyses were carried out with PLINK v1.9 (http://pngu.mgh.harvard.edu/purcell/plink/)^17^. For all groups of individuals, Hardy-Weinberg equilibrium was determined at a significance level of 0.01. Differences between allele and genotype frequencies in individuals were determined by χ^2^ test. Odds ratios (ORs) and 95% confidence intervals were calculated according to Woolf’s method. Meta-analyses were performed with inverse-variance method under a fixed-effects model. Heterogeneity of the ORs across cohorts was assessed using both Cochran’s Q test and inconsistency index (I^2^). The statistical power of our study was calculated by using Power Calculator for Genetic Studies 2006 (CaTS) software (http://www.sph.umich.edu/csg/abecasis/CaTS/)^18^, under an additive model. The statistical power of the study for determining significance at a *p* value of 0.05 is shown in Supplementary Table S1.

### Data availability

The complete datasets generated and analyzed during the current study are available from the corresponding author on reasonable request.

### Ethical use of human participants statement

All participants signed a written informed consent prior to their enrolment in the study for identified data to be published. All procedures were performed after signed informed consent was obtained from patients and in accordance with the local (Spanish National Research Council) ethics committee and the Declaration of Helsinki of 1975, as revised in 1983.

## Results

First, in order to evaluate the association between the *TNFSF13B* gene variant (BAFF-var) and susceptibility to RA, a comparative analysis of allelic and genotype frequencies in RA patients vs. healthy individuals was performed in the Spanish, Dutch and German populations. Results are shown in Table 1. The minor allele frequency (A) of *TNFSF13B* BAFF-var was higher in RA patients in the three populations included in this study. This difference was statistically significant in the Spanish subgroup (p = 0.015, OR = 1.21, 95% CI = 1.03–1.41), and suggestive associations were observed in the Dutch (p = 0.115, OR = 1.47, 95% CI = 0.91–2.36) and German (p = 0.228, OR = 1.39, 95% CI = 0.81–2.36) subgroups. In addition, a statistically significant *p* value was obtained in the meta-analysis of the three RA populations included in this study (p = 0.002, OR = 1.24, 95% CI = 1.10–1.38) with low heterogeneity levels (heterogeneity q value = 0.64, I^2^ = 0) (Table 1).

**Table 1:** Association analysis of the *TNFSF13B* BAFF-var in three independent RA cohorts and meta-analysis.

Subgroup (N)	Genotype (N)			MAF (%)	Allele test	
	A|A	A|GCTGT	GCTGT|GCTGT		P-value	OR [95% CI]^a^
**Spain**						
Controls (3,200)^b^	10	249	2941	4.20		
RA (4,429)	11	424	3994	5.03	0.015	1.21 [1.03–1.41]
**Netherlands**						
Controls (733)	0	28	705	1.91		
RA (890)	0	49	841	2.75	0.115	1.47 [0.91–2.36]
**Germany**						
Controls (470)^b^	0	19	451	2.02		
RA (899)	3	44	852	2.78	0.228	1.39 [0.81–2.36]
**Meta-analysis** ^c^						
Controls (4,403)						
RA (6,218)					0.002	1.24 [1.10–1.38]

Abbreviations: N: Number of individuals; MAF: Minor Allele Frequency; OR: Odds Ratio; CI: Confidence Interval.

^a^OR for the minor allele.

^b^Healthy controls were used as reference for RA and SLE patients in Spanish and German subgroups.

^c^Heterogeneity q value = 0.64, I^2^ = 0.

Secondly, to try to replicate the results previously published by Steri *et al*.^5^, a comparative analysis of allelic and genotype frequencies in SLE patients vs. healthy individuals from the Spanish and German populations was also performed (Table 2). The minor allele frequency (A) in *TNFSF13B* BAFF-var was statistically significantly higher in SLE patients than in controls in both, the Spanish (p = 0.0001, OR = 1.41, 95% CI = 1.14–1.74) and German (p = 0.030, OR = 1.86, 95% CI = 1.05–3.28) subgroups. Meta-analysis of the two SLE populations included in this study revealed a statistically significant *p* value (p = 0.0002, OR = 1.46, 95% CI = 1.09–2.32) with low heterogeneity levels (heterogeneity q value = 0.37, I^2^ = 0) (Table 2).

**Table 2:** Association analysis of the *TNFSF13B* BAFF-var in two independent SLE cohorts and meta-analysis.

Subgroup (N)	Genotype (N)			MAF (%)	Allele test	
	A|A	A|GCTGT	GCTGT|GCTGT		P-value	OR [95% CI]^a^
**Spain**						
Controls (3,200)^b^	10	249	2941	4.20		
SLE (1,160)	9	117	1034	5.81	0.001	1.41 [1.14–1.74]
**Germany**						
Controls (470)^b^	0	19	451	2.02		
SLE (460)	1	32	427	3.69	0.030	1.86 [1.05–3.28]
**Meta-analysis** ^c^						
Controls (4,403)						
SLE (6,218)					0.0002	1.46 [1.09–2.32]

Abbreviations: N: Number of individuals; MAF: Minor Allele Frequency; OR: Odds Ratio; CI: Confidence Interval.

^a^OR for the minor allele.

^b^Healthy controls were used as reference for RA and SLE patients in Spanish and German subgroups.

^c^Heterogeneity q value = 0.37, I^2^ = 0.

## Discussion

We confirm the recently reported association of the *TNFSF13B* BAFF-var with SLE in two independent populations of Spanish and German origin. In addition, this is the first time that evidence is provided of an association between *TNFSF13B* BAFF-var and RA. These findings, together with the data reported by Steri *et al*.^5^, suggest that *TNFSF13B* BAFF-var is a common genetic risk factor for autoimmunity.

SLE and RA mice models showed increased serum BAFF levels, and BAFF blockade reduced diseases manifestations^19,20^. Nevertheless, although clinical trials are being performed on BAFF in RA and SLE patients, only moderate results are emerging. An example of these modest results is the BAFF-inhibitor drug belimumab, which has been found to be only moderately effective in a small number of patients with RA and SLE^8,21^. Because RA and SLE are heterogeneous diseases with different biological subsets, the efficacy of different drugs could be divergent in specific patient subgroups. Therefore, our new findings could help in the advance of RA therapies, as patients stratified by *TNFSF13B* BAFF-var status may show a differential benefit from anti-BAFF therapies. In this sense, a recent study showed that a *TNFSF13B* genetic variant influenced response to B-cell targeted therapy (rituximab) in patients with RA^22^. Furthermore, B-cell-depleting therapies in patients carrying *TNFSF13B* BAFF-var would have a weaker effect due to a rapid resurgence of memory B cells induced by high soluble BAFF levels, which could increase the risk of inadequate response and/or relapse^23^.

Genome-wide association studies (GWAS) have identified multiple risk genetic factors associated with human autoimmune disease. Of note, GWAS are designed to detect only common genetic variants (>5%); however, low-frequency and rare variants may represent an important component of autoimmune risk and provide a key insight into both novel and previously implicated immunological pathways that are disrupted in autoimmune diseases^24^. Thus, large genetic studies have identified low frequency variants with relevant functional significance associated with autoimmune diseases (e.g. *TREX1* in SLE^25^ and *TYK2* in several autoimmune diseases^26,27^). Our findings support the important role of rare variants, such as *TNFSF13B* BAFF-var, in understanding the unknown mechanisms of autoimmunity.

For the first time, our results showed that allele A of *TNFSF13B* BAFF-var was associated with the risk of RA in the Spanish population. Trends of association were observed in the Dutch and German RA cohorts. This could be due to a low statistical power as the Dutch and the German cohorts sample sizes are relatively small as compared to the Spanish cohort, as well as the minor allele frequency is less common in northern Europe (2.02% in Germany and 1.91% in the Netherlands) than in southern Europe (4.2% in Spain). This north-south gradient in the minor allele frequency of *TNFSF13B* BAFF-var has been previously reported, with higher frequencies observed in southern Europe (5.7% in Italy and 4.9% in Spain) than in northern Europe (1.8% in United Kingdom and Sweden)^5^. However, the three RA cohorts showed higher levels of allele A in cases than in controls and statistically significant association was observed in the meta-analysis. Thus, further studies involving larger cohorts would be necessary to elucidate if this variant is associated with autoimmune risk in northern populations.

Regarding the SLE replication study, our results confirm the association of allele A of *TNFSF13B* BAFF-var with the risk of SLE in the Spanish and German populations. The association of the variant was found to be stronger in SLE than in RA and, in both cases, the allele A acts as a risk factor.

GWAS have revealed a significant shared genetic component in autoimmunity^28,29^. The association of *TNFSF13B* BAFF-var with RA and SLE observed in our study extends the number of overlapping genetic risk factors across different autoimmune diseases. In this regard, performing further studies to elucidate the specific association of *TNFSF13B* BAFF-var with other autoimmune diseases would be of great interest as new therapy approaches involving BAFF could emerge.

In conclusion, our results suggest that *TNFSF13B* BAFF-var plays an important role in susceptibility to RA, and confirm the association of this variant with SLE.

## Electronic supplementary material

Supplementary Information
