# Supplementary material for: Association of a rare variant of the TNFSF13B gene with susceptibility to Rheumatoid Arthritis and Systemic Lupus Erythematosus
Source: Sci Rep. 2018 May 29;8:8195. doi: 10.1038/s41598-018-26573-4 (PMC5974315; doi:10.1038/s41598-018-26573-4)
Supplement: Supplementary file 1 — Supplementary Information [file 41598_2018_26573_MOESM1_ESM.pdf]

Association of a rare variant in *TNFSF13B* gene with susceptibility to Rheumatoid Arthritis and Systemic Lupus Erythematosus

David González-Serna, Lourdes Ortiz-Fernández, Sofía Vargas, Antonio García, Enrique Raya, Benjamín Fernández-Gutierrez, Francisco Javier López-Longo, Alejandro Balsa, Isidoro González-Álvaro, Javier Narvaez, Carmen Gómez-Vaquero, José Mario Sabio, Rosa García-Portales, María Francisca González-Escribano, Carles Tolosa, Patricia Carreira, Lambertus Kiemeney, Marieke J.H. Coenen, Torsten Witte, Matthias Schneider, Miguel Ángel González-Gay, Javier Martín*

Supplementary Table S1. Statistical power calculation performed using Power Calculator for Genetic Studies 2006 (CaTS) software (http://www.sph.umich.edu/csg/abecasis/CaTS/)

Statistical Power Calculation

	RA from Spain	RA from the Netherlands	RA from Germany	SLE from Spain	SLE from Germany
OR = 1.5	100%	46%	36%	93%	28%
OR = 1.4	99%	33%	26%	84%	20%
OR = 1.3	95%	21%	17%	61%	14%
OR = 1.2	75%	13%	11%	34%	9%

OR: Odds ratio
